# The relationship between executive functions and fluid intelligence in schizophrenia

**DOI:** 10.3389/fnbeh.2014.00046

**Published:** 2014-02-24

**Authors:** María Roca, Facundo Manes, Marcelo Cetkovich, Diana Bruno, Agustín Ibáñez, Teresa Torralva, John Duncan

**Affiliations:** ^1^Neuropsychology Research Department, Institute of Cognitive Neurology (INECO)Buenos Aires, Argentina; ^2^Laboratory of Cognitive and Social Neuroscience (LaNCyS), UDP-INECO, Foundation Core on Neuroscience (UIFCoN), Diego Portales UniversitySantiago, Chile; ^3^Neuropsychology Department, Institute of Neurosciences Favaloro UniversityBuenos Aires, Argentina; ^4^Universidad Autónoma del CaribeBarranquilla, Colombia; ^5^MRC Cognition and Brain Sciences UnitCambridge, UK; ^6^Department of Experimental Psychology, University of OxfordOxford, UK

**Keywords:** executive function, fluid intelligence, schizophrenia, frontal lobe, multitasking, decision making

## Abstract

An enduring question is unity vs. separability of executive deficits resulting from impaired frontal lobe function. In previous studies, we have asked how executive deficits link to a conventional measure of fluid intelligence, obtained either by standard tests of novel problem-solving, or by averaging performance in a battery of novel tasks. For some classical executive tasks, such as the Wisconsin Card Sorting Test (WCST), Verbal Fluency, and Trail Making Test B (TMTB), frontal deficits are entirely explained by fluid intelligence. However, on a second set of executive tasks, including tests of multitasking and decision making, deficits exceed those predicted by fluid intelligence loss. In this paper we discuss how these results shed light on the diverse clinical phenomenology observed in frontal dysfunction, and present new data on a group of 15 schizophrenic patients and 14 controls. Subjects were assessed with a range of executive tests and with a general cognitive battery used to derive a measure of fluid intelligence. Group performance was compared and fluid intelligence was introduced as a covariate. In line with our previous results, significant patient-control differences in classical executive tests were removed when fluid intelligence was introduced as a covariate. However, for tests of multitasking and decision making, deficits remained. We relate our findings to those of previous factor analytic studies describing a single principal component, which accounts for much of the variance of schizophrenic patients' cognitive performance. We propose that this general factor reflects low fluid intelligence capacity, which accounts for much but not all cognitive impairment in this patient group. Partialling out the general effects of fluid intelligence, we propose, may clarify the role of additional, more specific cognitive impairments in conditions such as schizophrenia.

## Introduction

Although the efforts of multiple disciplines have brought substantial advances in the comprehension on frontal lobe functioning, much remains unclear in how this brain region participates in the organization of effective behavior. In general, prefrontal cortex (PFC) is supposed to participate in distributed brain circuits underlying “executive” functions, broadly conceived as processes that organize and control cognitive activity. Often, however, theoretical frameworks of frontal and executive functions give little detailed explanation for the wide variety of deficits produced by frontal lobe lesions. Furthermore, advances in cognitive neuroscience have rarely translated into improved clinical analysis and management of pathologies affecting frontal lobe functions.

In this paper we discuss previous results coming from basic and clinical neuroscience in order to shed light on the diverse clinical phenomenology observed in frontal dysfunction. Additionally, we present new data on a group of schizophrenic patients, aiming to demonstrate how basic cognitive neuroscience and clinical neuropsychology can converge to explain the broad cognitive deficit observed in this population. In particular, our results cast light on the balance between global cognitive deficit and specific functional impairments.

Commonly, different regions of the PFC are supposed to participate in different kinds of executive function. One important framework, for example, proposes functions such as planning, monitoring, energizing, switching, and inhibition (Stuss et al., 2002; Stuss, 2007). Though some kind of functional specialization in different regions of PFC seems certain, its detailed nature remains elusive. In recent studies addressing a variety of frontal lobe pathologies, we have proposed a framework that combines elements of broad cognitive impairment—important in many different tasks and contexts—with additional, more specific deficits (Roca et al., 2010, 2012, 2013).

One motivation for our proposal comes from functional imaging. In some frontal regions, similar activity is seen while performing very diverse cognitive tasks (Cabeza and Nyberg, 1997, 2000; Duncan and Owen, 2000). This finding of activity in many different tasks suggests what we have called a multiple-demand or MD system, important in organization of many kinds of behavior (Duncan, 2005, 2010). MD activity is commonly seen in circumscribed regions of lateral frontal cortex, dorsomedial frontal cortex, and anterior insula, with accompanying similar activity around the intraparietal sulcus (Fedorenko et al., 2012). A hint of why such extensive brain regions could be activated in so many different kinds of tasks comes from single cell electrophysiology studies in monkeys. Neurons of lateral PFC adapt their properties to task context, coding the specific information required in current behavior (Rao et al., 1997; Freedman et al., 2001). In this way they may represent a general neural resource, adapting to contribute to many different kind of task. Elsewhere we have suggested that the core function of the MD system is to organize complex cognition into a structured series of attentional episodes, assembling the selected contents of each episode, and managing transitions from one episode to the next (Duncan, 2013).

Closely related to these findings is the role of frontal functions in general intelligence or Spearman's *g*. Based on the universal positive correlations typically found between different cognitive tests, Spearman (1904, 1927) proposed that a general or *g* factor contributes to all cognitive activities. On this theory, one way to measure *g* is simply to average performance across a diverse set of tests (Spearman, 1927), the approach taken in traditional IQ tests such as the WAIS (Wechsler, 1997). Another is to find single tests that, on their own, correlate strongly with the average across multiple tests. The best single tests, often called tests of “fluid intelligence,” call for novel problem-solving using visual, verbal or other materials (e.g., Raven et al., 1988). Common activity in MD regions for many kinds of behavior suggests a plausible basis for *g*, and in line with this, frontal lesions impair performance on classic fluid intelligence tests (Duncan et al., 1995). This may be especially so when lesions affect MD regions (Woolgar et al., 2010). Strong MD activity is also seen while performing fluid intelligence tests (Prabhakaran et al., 1997; Esposito et al., 1999; Duncan et al., 2000; Bishop et al., 2008). In line with our proposals concerning MD function, a salient requirement of fluid intelligence tests is that complex problems must be divided into simpler parts, calling for a novel structure of attentional episodes (Duncan, 2013).

For some years, our group has been investigating the clinical relevance of these findings in different frontal pathologies. To this end, we have investigated fluid intelligence tests and executive deficits in several clinical conditions, including frontal lobe lesions (Roca et al., 2010), Parkinson's Disease (Roca et al., 2012), and Frontotemporal Dementia (Roca et al., 2013). In particular, we have asked how much fluid intelligence loss contributes to deficits in classical executive tasks. Consistently, our results (Roca et al., 2010, 2012, 2013) have shown the same picture. For some classical executive tasks, such as the Wisconsin Card Sorting Test (WCST; Nelson, 1976), Verbal Fluency (Benton and Hamsher, 1976), and Part B of the Trail Making Test (TMTB; Partington and Leiter, 1949), executive deficits are entirely explained by fluid intelligence. Once a measure of fluid intelligence is partialled out, no differences between patients and normal controls remain (for an exception in the case of Verbal Fluency see Robinson et al., 2012). These results suggest that frontal deficits in such tasks are associated with rather general cognitive processes rather than the specific content of individual tests. Other executive tests, however, show a different picture, with clinical deficits not explained by fluid intelligence. Most consistently falling in this group are multitasking tests, which assess the ability to select and maintain higher order internal goals while other sub-goals are being performed, and tests of social cognition. In these tasks, our data suggest that deficits are related to damage outside the classical MD regions, in particular the most anterior part of the frontal cortex, known as the frontal pole or APFC (Roca et al., 2010, 2011). Intriguingly, we have found mixed results with a further test, the Iowa Gambling Test (IGT) of probabilistic decision-making. Previous data suggest a multicomponent test, with prominent contributions from ventromedial frontal cortex but also other frontal areas (Bechara et al., 2000; Manes et al., 2002), and we found deficits beyond those explained by fluid intelligence only for pathology with strong ventromedial involvement (Roca et al., 2013).

Based on these findings, we propose a new way to dissociate executive impairments in frontal lobe pathology. While damage to MD regions affects fluid intelligence, impairing performance on many classical executive tests, other regions of damage produce deficits that go beyond those explained by fluid intelligence and that cannot be captured by classical executive tests. Our approach bears on one of the oldest and most central problems in clinical neurology. Some patients with frontal lobe dysfunction present obvious cognitive and behavioral deficits, even if they perform remarkably well in many neuropsychological examinations. This so-called “frontal lobe mystery” has been at the center of clinical neuropsychology debates and has been particularly described in patients with damage involving APFC regions (Burgess, 2000).

Here, we apply this framework to the question of global cognitive deficit vs. specific executive impairments in schizophrenia. As in various psychiatric conditions, cognitive deficits in schizophrenia have been commonly explained in terms of frontal dysfunction (e.g., Lewis and González-Burgos, 2008; Lewis, 2012), and in anatomical terms, a range of frontal abnormalities have been described (Ellison-Wright et al., 2008; Haukvik et al., 2013). Importantly, some recent factor analytic studies have described a single principal component that accounts for much of the variance of patients' cognitive performance (e.g., Dickinson et al., 2006, 2008; Keefe et al., 2006; Harvey et al., 2011, 2013). For example, Dickinson et al. (2008) used structural equation modeling to show that approximately 63% of the schizophrenia diagnosis-related variance in cognitive performance was mediated through a single general factor. Using a very large sample size (*n* = 1493), Keefe et al. (2006) compared the efficacy of various competing models in their ability to account for the structure of cognitive deficits in schizophrenia. Their exploratory principal component analysis showed that a unifactorial structure, which accounted for 45% of the variance of patients' cognitive performance, was the best fitting model to describe patients' cognitive functioning. Deficits in fluid intelligence tests have also been reported in this population (e.g., Caspi et al., 2003; Zanello et al., 2006; Johnson et al., 2013). Accordingly, a general “*g* like” deficit seems to explain much of the cognitive deficit in this patient group.

In parallel to this factor analytic work, neuropsychological studies of schizophrenia show deficits in a range of executive tests, including the WCST, multitasking and tests of social cognition (e.g., Liddle and Morris, 1991; Frith et al., 1992; Thoma and Daum, 2005; Thoma et al., 2007; Kim et al., 2009; Egan et al., 2011; Banno et al., 2012; Cochrane et al., 2012; Erol et al., 2012; Baez et al., 2013; Fond et al., 2013; Sánchez-Torres et al., 2013). Again there is evidence for some link to fluid intelligence. For example, as part of a broader study aimed at describing the pattern of relationships between measures of cognitive performance and symptom subtypes in schizotypy and schizophrenia, Cochrane et al. (2012) examined both fluid intelligence (measured using the Matrices test of the Wechsler Intelligence Scale; Wechsler, 1997) and Verbal Fluency. Low fluency scores were associated with the negative factor of a clinical interview schedule (SANS), and this association was reduced when fluid intelligence was introduced as a covariate. Also, executive functions and fluid intelligence impairments were associated variables of the same latent factor using structural equation models in schizophrenia patients, as well as in their first degree relatives and other psychiatric disorders (Ibáñez et al., 2013).

Here we test the prediction that a broad, “*g* like” cognitive decline accounts for some but not all of the executive impairment in schizophrenia. As in our previous work, we ask how strongly executive deficits remain once fluid intelligence is introduced as a covariate. We predict that, for some classical executive tests, impairments in schizophrenia will be fully explained by reduced fluid intelligence. For other tests—here including multitasking and probabilistic decision-making—we predict that deficits will remain even once the effects of fluid intelligence are removed.

## Materials and methods

### Participants

Fifteen patients with a diagnosis of schizophrenia according to DSM-IV criteria were recruited for a broader ongoing study of schizophrenia at the Institute of Cognitive Neurology (INECO). The Positive and Negative Syndrome Scale (PANSS) was used for measuring symptom severity. All patients gave informed consent and underwent a detailed examination of their psychiatric and neuropsychological profile, supported by EEG, SPECT, and MRI as needed for diagnosis. All patients were receiving antipsychotic treatment and showed stable psychotic symptoms for a period of at least 8 weeks, over which no change in medication dose or type was indicated. Mean (*SD*) age for the patient population was 36.7 (8.6). Mean PANSS total score was 68.0 (16.9).

Fourteen healthy controls were randomly recruited from a larger pool of volunteers who had neither a history of abuse of recreational drugs nor a family history of neurodegenerative or psychiatric disorders. Mean (*SD*) age for the control group was 42.6 (14.7) years. All participants in the study were examined to assure they had no comorbidity with other psychiatric or neurological disorders.

### Neuropsychological assessment

#### Wisconsin card sorting test (Nelson, 1976)

For the WCST we used Nelson's modified version of the standard procedure. Cards varying on three basic features—color, shape, and number of items—must be sorted according to each feature in turn. The participant's first sorting choice becomes the correct feature, and once a criterion of six consecutive correct sorts is achieved, the subject is told that the rules have changed, and cards must be sorted according to a new feature. After all three features have been used as sorting criteria, subjects must cycle through them once again in the same order as they did before. Each time the feature is changed, the next must be discovered by trial and error. Score was total number of categories achieved before completing a maximum of 48 cards.

#### Verbal fluency (Benton and Hamsher, 1976)

In Verbal Fluency tasks, the subject generates as many items as possible from a given category. We used the standard Argentinean phonemic version (Butman et al., 2000), asking subjects to generate words beginning with the letter P in a one-minute block. Score was the total number of correct words generated.

#### Trail making test B (Partington and Leiter, 1949)

The Trail Making Test consists of two parts. In the present study part B was administered (TMTB). In this test the subject is required to draw lines sequentially connecting 13 numbers and 12 letters distributed on a sheet of paper. Letters and numbers are encircled and must be connected alternately in increasing/alphabetical order (i.e., 1, A, 2, B, 3, C, etc.). Score was the total time (s) required to complete the task, given a negative sign so that high scores meant better performance.

#### Hotel task (Manly et al., 2002; Torralva et al., 2009)

The task comprised five primary activities related to running a hotel (compiling bills, sorting coins for a charity collection, looking up telephone numbers, sorting conference labels, proofreading). The materials needed to perform these activities were arranged on a desk, along with a clock that could be consulted by removing and then replacing a cover. Subjects were told to try at least some of all five activities during a 15 min period, so that, at the end of this period, they would be able to give an estimate of how long each task would take to complete. It was explained that time was not available to actually complete the tasks; the goal instead was to ensure that every task was sampled. Subjects were also asked to remember to open and close the hotel garage doors at specified times (open at 6 min, close at 12 min), using a coloured button. Of the several scores possible for this task, we used time allocation: for each primary task we assumed an optimal allocation of 3 min, and measured the summed total deviation (in seconds) from this optimum. Total deviation was given a negative sign so that high scores meant better performance.

#### Iowa gambling task (Bechara et al., 2000)

In the IGT, subjects are required to pick cards from four decks and receive rewards and punishments (winning and losing abstract money) depending on the deck chosen. Two “risky” decks yield greater immediate wins but very significant occasional losses. The other two “conservative” decks yield smaller wins but negligible losses that result in net profit over time. Subjects make a series of selections from these four available options, from a starting point of complete uncertainty. Reward and punishment information acquired on a trial by trial basis must be used to guide behavior toward a financially successful strategy. Normal subjects increasingly choose conservative decks over the 100 trials of the task. Our score was the total number of conservative minus risky choices.

#### General test battery (GTB)

All participants were also assessed with a general test battery used to derive a measure of fluid intelligence. As noted above, fluid intelligence can be measured either using a standard psychometric test such as Raven's Matrices (Raven et al., 1988) or simply by averaging performance on a diverse battery of novel tasks; in practice, these two approaches give largely similar results, and we used the latter method here. The general test battery included Forward Digit Span (Wechsler, 1997), the Rey Auditory Verbal Learning Test (Rey, 1941), the Rey Complex Figure Test (Rey, 1941), and Trail Making Test A (Partington and Leiter, 1949). For this set of tests, principal component analysis produced a first component accounting for 55.61% of the total variance. Loadings on this component were moderate to high for all tests (range = 0.45–0.88). The general or *g* score for each participant (*g*GTB) was defined as the score on this first principal component.

## Results

Groups were matched for age and premorbid IQ as measured with the Word Accentuation Test (Burin et al., 2000). For all tests, the mean scores of each group are shown in Table 1. For all cognitive tasks, two-tailed *t*-tests were used to compare patients and controls. As expected, the schizophrenic group was significantly impaired on all tests, including the classical executive tests [WCST: *t*_(27)_ = −3.31, *p* < 0.01; Verbal Fluency: *t*_(27)_ = −2.86, *p* < 0.01; TMTB *t*_(27)_ = 2.48, *p* = 0.02] and the tests of multitasking and decision making [Hotel: *t*_(27)_ = 3.34, *p* < 0.01; IGT: *t*_(27)_ = −3.47, *p* < 0.01]. For *g*GTB significant differences between groups were also found, [*t*_(27)_ = −4.33, *p* < 0.01].

**Table 1:** **Patient and control scores and significance of group differences, before and after *g*GTB was introduced as a covariate**.

	**Controls mean (*SD*)**	**Schizo mean (*SD*)**	**Average within groups correlations with *g*GTB**	**Main effect of group before *g*GTB was introduced as a covariate (*df* = 2, 27)**	**Main effect of group after *g*GTB was introduced as a covariate**
Age	42.6 (14.7)	36.67 (6.6)	–	*P* = 0.2	–
WAT	36.6 (5.4)	32.5 (8.3)	–	*P* = 0.12	–
WCST	5.5 (0.9)	3.7 (1.9)	0.44	***P* < 0.01**	*P* = 0.27
Verbal fluency	17.4 (6.0)	11.7 (4.6)	0.51	***P* < 0.01**	*P* = 0.56
TMTB	−94.4 (74.2)	−164.7 (78.3)	0.68	***P* = 0.02**	*P* = 0.64
Hotel task	−288.9 (133)	−518.7 (227.7)	0.05	***P* < 0.01**	***P* = 0.03**
IGT	30.1 (27.4)	−8.9 (32.7)	0.11	***P* < 0.01**	***P* < 0.01**

*gGTB, g score for each participant derived from the general cognitive battery; WAT, Word Accentuation Test; WCST, Wisconsin Card Sorting Test; TMTB, Trail Making Test part B; IGT, Iowa Gambling Task. Statistically significant differences (*p* < 0.05) are marked in bold*.

**Figure 1:**
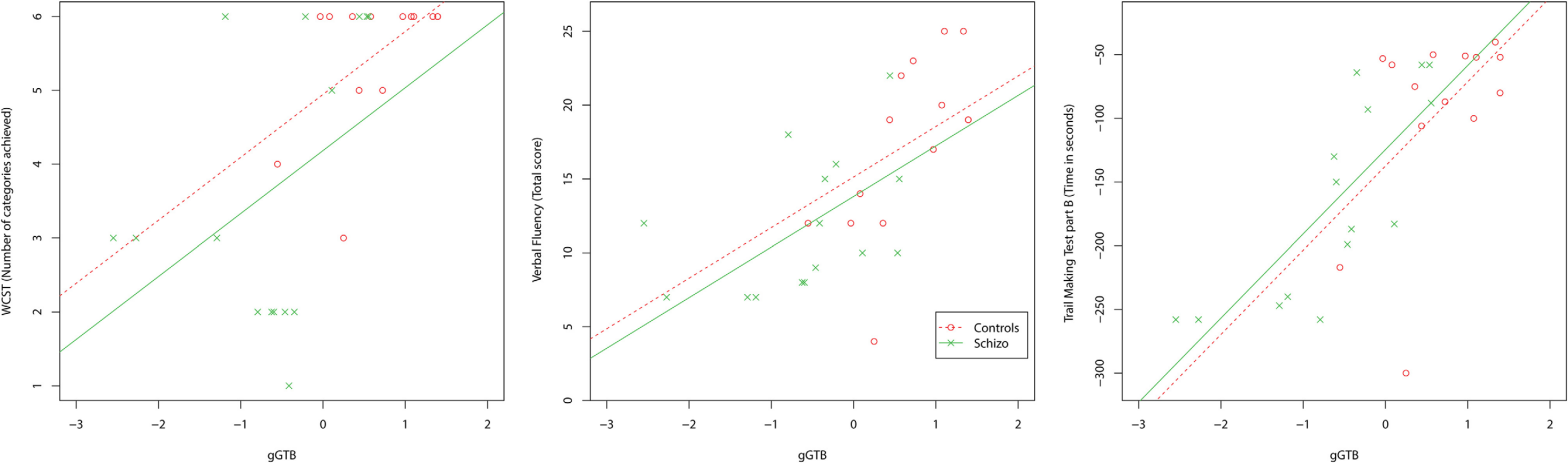
**Scatterplots relating *g*GTB to the three classical executive tests**. Regression lines reflect the average within-group association of the two variables, as determined by ANCOVA, constrained to have the same slope across groups.

Scatterplots relating *g*GTB to the three classical executive tests are shown in Figure 1. For all classical executive tests, scores were heavily dependent on *g*GTB, and once this influence was removed by ANCOVA, group differences were no longer significant (Table 1). Regression lines in Figure 1 come from the standard ANCOVA model, reflecting the average within-group association of the two variables and constrained to have the same slope across groups. As calculated from the corresponding variance terms of the ANCOVA, average within-group correlations with *g*GTB were 0.44 for WCST, 0.51 for Verbal Fluency, and 0.68 for TMTB.

**Figure 2:**
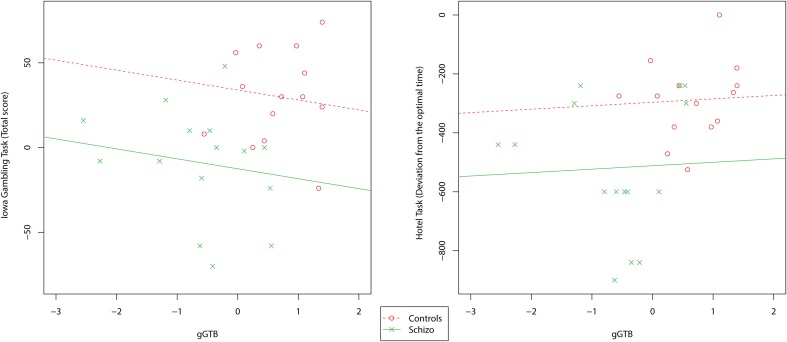
**Scatterplots relating *g*GTB to the multitasking and decision-making tests**. Regression lines reflect the average within-group association of the two variables, as determined by ANCOVA, constrained to have the same slope across groups.

Scatterplots relating *g*GTB to the other executive tests are shown in Figure 2. For these tests results were very different. Scores were barely related to *g*GTB, with average within-group correlations of 0.05 for the Hotel Task and 0.11 for the IGT. In both cases using ANCOVA to remove the influence of *g*GTB left significant group differences (Table 1).

## Discussion

Though it seems certain that the frontal lobes contribute to multiple cognitive functions, it remains unclear how these functions should be separated and defined. Recently, we have proposed a novel parcellation, based on the role of fluid intelligence. For one set of executive tests, deficits in a variety of neurological and neuropsychiatric conditions are entirely explained by loss of fluid intelligence. For these tests, once the effects of fluid intelligence are partialled out, no clinical deficit remains. These deficits, we propose, are explained by damage to the distributed frontoparietal MD system, important in constructing any cognitive activity from a structured series of attentional episodes. For other tests, in contrast, deficits remain even after effects of fluid intelligence are removed. For one group of tests, including tests of multitasking and social cognition, deficits may relate to impaired function in the APFC. For the IGT, deficits not explained by fluid intelligence may reflect the value-based decision-making functions of ventromedial frontal cortex (Bechara et al., 2000). More broadly, we propose that removing the effects of fluid intelligence may clarify relations between other more specific executive impairments and frontal regions outside the MD system.

Here, we apply this novel parcellation of frontal lobe functions to the case of schizophrenia. Consistent with our results in multiple neuropsychiatric conditions with frontal involvement (Roca et al., 2010, 2012, 2013), fluid intelligence proves to be a substantial contributor to many executive deficits in this disease. For some widely used executive tasks, including the WCST, Verbal Fluency, and TMTB, patients' deficits are entirely explained by individual fluid intelligence scores. Once fluid intelligence is partialled out, no differences between patients and normal controls remain. For a second set of executive tasks, however, deficits persist even after fluid intelligence is statistically controlled. In the present data, such results were seen for tests of multitasking (Hotel Task) and decision-making (IGT).

Our results are consistent with investigations suggesting that a single factor accounts for much of the cognitive impairment in schizophrenia (Dickinson et al., 2004; Keefe et al., 2006; Dickinson and Harvey, 2009; Dickson et al., 2011). Following our previous findings, we propose that this general factor links closely to standard measures of fluid intelligence, suggesting impaired function in the distributed, frontoparietal MD system. Importantly, our results show that this is only a part of the picture. Some other deficits observed in this population exceed this general deficit, arguing in favor of specific functional impairments that are not so closely related to *g*. In this investigation, we show that the multitasking and decision making deficits evidenced in schizophrenic patients represent specific cognitive deficits, rather than general ones.

Our multitasking results have important implications for the overall functional architecture of cognitive control in schizophrenia. Neuropsychological (Roca et al., 2011) and neuroimaging studies (e.g., Burgess et al., 2007; Badre and D'Esposito, 2009) have related multitasking with the correct functioning of APFC. In this regard, our results are consistent with recent studies which have proposed that in schizophrenic patients, the organization of cognitive control follows a rostro-caudal organization within the PFC (Barbalat et al., 2011), with APFC being at the top of a frontal processing hierarchy (Koechlin et al., 2003; Badre and D'Esposito, 2007).

Decision making deficits have also been described in schizophrenia (e.g., Kim et al., 2009; Fond et al., 2013). Classically, impairments on the IGT have been related to ventromedial frontal damage (Bechara et al., 2000), but deficits also follow lesions in other frontal regions, suggesting a multicomponent test (Manes et al., 2002). In patients with focal frontal lesions, rarely extending to ventromedial cortex, we found IGT deficits to be fully explained by fluid intelligence (Roca et al., 2010). In Frontotemporal Dementia, a disease with a strong ventromedial component, the IGT fell into the alternative group of tests, with deficits that remained even once effects of fluid intelligence were removed (Roca et al., 2013). The present results suggest that, in schizophrenia also, decision making deficits represent a specific rather than a general impairment, possibly reflecting ventromedial PFC dysfunction.

The fact that general and specific deficits are found in schizophrenia—with several classical executive deficits falling in the first group and multitasking and decision making deficits falling in the second—clarifies the understanding of cognitive deficits in this pathology. Coincidently with the widespread pathology of this disease, the associated cognitive deficits seem to represent a multicomponent cognitive dysfunction, but with a strong *g*-like element.

We believe that our results raise serious methodological issues related to testing procedures used in patients with schizophrenia. Both in clinical practice and research with this patient group, many different tests are used as measures of frontal dysfunction. Deficits in the WCST (e.g., Egan et al., 2011; Banno et al., 2012; Sánchez-Torres et al., 2013), Verbal Fluency (e.g., Liddle and Morris, 1991; Frith et al., 1992; Cochrane et al., 2012), and TMTB (e.g., Chan et al., 2006; Erol et al., 2012; Sánchez-Torres et al., 2013) have been consistently reported. Impairments in such tasks are often interpreted with close reference to specific test content. In the WCST, for example, deficits are ascribed to impaired switching of cognitive set (Milner, 1963) while in verbal fluency, the frontal impairment (Benton, 1968) is commonly interpreted as a failure in spontaneous generation of new search strategies. However, in the present study we show that deficits in these tests are entirely explained by a fluid intelligence loss. For these tests, it seems likely that specific test content is unimportant for interpreting the schizophrenic deficit. Instead, deficits reflect a broad cognitive disorganization affecting many different kinds of complex cognition. Equally significant are the implications for identifying deficits that go beyond those explained by fluid intelligence, including deficits in multitasking and probabilistic decision-making. Given the importance of fluid intelligence in tasks of many kinds, removing its affects statistically may be important in allowing other, more specific deficits to be uncovered.

Our results have important clinical implications, both for the use of appropriate assessment tools and for the implementation of adequate rehabilitation strategies in schizophrenia. In our view, an optimal neuropsychological assessment in this population should include a fluid intelligence test in order to capture the general factor affected in the disease. Though the present results are limited by the particular group of executive tests employed, the role of fluid intelligence in predicting executive deficits is likely to be widespread. A recent study, for example, shows that a conventional intelligence test explains most or all frontal deficits in the Delis Kaplan Executive Function System (D-KEFS; Barbey et al., 2013). A fluid intelligence test must be supplemented, however, by tasks that are able to detect additional deficits, including multitasking and decision making tasks. From a rehabilitation perspective, it can be inferred that strategies targeting fluid intelligence deficits (e.g., Jaeggi et al., 2008; Jaušovec and Jaušovec, 2012) should be separated from strategies targeting specific multitasking and decision-making deficits (e.g., Manly et al., 2002). Further investigations should explore the differential impact of fluid intelligence and other frontal deficits in patients' daily living and quality of life.

In the present paper we propose an approach that can explain, at least in part, some of the variety of deficits associated with frontal functions. The broad approach addresses clinical findings in diverse pathologies with frontal dysfunction and, in particular, we demonstrated how this model could explain previous results coming from factor analytic studies in schizophrenia. We believe that our approach represents a step forward toward the required trans-disciplinary unification of theory and practice in the investigation of frontal lobe function. Here, we show how results coming from basic and clinical neuropsychology can converge to address neurological and neuropsychiatric conditions related to frontal functioning, particularly in schizophrenia.

## Author contributions

All authors participated in the writing of the manuscript. María Roca designed the study, completed the research, and led the writing of the manuscript. Diana Bruno, Teresa Torralva, Marcelo Cetkovich, and Agustín Ibáñez supervised data collection and helped with the writing. Facundo Manes and John Duncan supervised the design and writing of the manuscript.

### Conflict of interest statement

The authors declare that the research was conducted in the absence of any commercial or financial relationships that could be construed as a potential conflict of interest.
